# Allergic and other adverse reactions to drugs used in anesthesia and surgery

**DOI:** 10.1007/s44254-023-00018-2

**Published:** 2023-06-14

**Authors:** Brian A. Baldo

**Affiliations:** 1 Molecular Immunology Unit, Kolling Institute of Medical Research, Royal North Shore Hospital of Sydney, St Leonards, Australia; 2 Department of Medicine, University of Sydney, Sydney, NSW Australia; 3 Lindfield, Australia

**Keywords:** Adverse drug reactions, Drug allergy, Perioperative drug reactions, Genetic factors and adverse drug reactions, Adverse drug reactions in anesthesia and surgery, Pharmacogenomics of adverse drug reactions, MRGPRX2-mediated adverse reactions

## Abstract

The list of drugs patients may be exposed to during the perioperative and postoperative periods is potentially extensive. It includes induction agents, neuromuscular blocking drugs (NMBDs), opioids, antibiotics, sugammadex, colloids, local anesthetics, polypeptides, antifibrinolytic agents, heparin and related anticoagulants, blue dyes, chlorhexidine, and a range of other agents depending on several factors related to individual patients’ clinical condition and progress in the postoperative recovery period. To avoid poor or ultrarapid metabolizers to a particular drug (for example tramadol and codeine) or possible adverse drug reactions (ADRs), some drugs may need to be avoided during or after surgery. This will be the case for patients with a history of anaphylaxis or other adverse events/intolerances to a known drug. Other drugs may be ceased for a period before surgery, e.g., anticoagulants that increase the chance of bleeding; diuretics for patients with acute renal failure; antihypertensives relative to kidney injury after major vascular surgery; and serotonergic drugs that together with some opioids may rarely induce serotonin toxicity. Studies of germline variations shown by genotyping and phenotyping to identify a predisposition of genetic factors to ADRs offer an increasingly important approach to individualize drug therapy. Studies of associations of human leukocyte antigen (HLA) genes with some serious delayed immune-mediated reactions are ongoing and variations of drug-metabolizing cytochrome CYP450 enzymes, P-glycoprotein, and catechol-*O*-methyltransferase show promise for the assessment of ADRs and non-responses to drugs, particularly opioids and other analgesics. Surveys of ADRs from an increasing number of institutions often cover small numbers of patients, are retrospective in nature, fail to clearly identify culprit drugs, and do not adequately distinguish immune-mediated from non-immune-mediated anaphylactoid reactions. From the many surveys undertaken, the large list of agents identified during and after anesthesia and surgery are examined for their ADR involvement. Drugs are classified into those most often involved, (NMBD and antibiotics); drugs that are becoming more frequently implicated, namely antibiotics (particularly teicoplanin), and blue dyes; those becoming less frequently involved; and drugs more rarely involved in perioperative, and postoperative adverse reactions but still important and necessary to keep in mind for the occasional potential sensitive patient. Clinicians should be aware of the similarities between drug-induced true allergic type I IgE/FcεRI- and pseudoallergic MRGPRX2-mediated ADRs, the clinical features of each, and their distinguishing characteristics. Procedures for identifying MRGPRX2 agonists and diagnosing and distinguishing pseudoallergic from allergic reaction mechanisms are discussed.

**Graphical Abstract:**

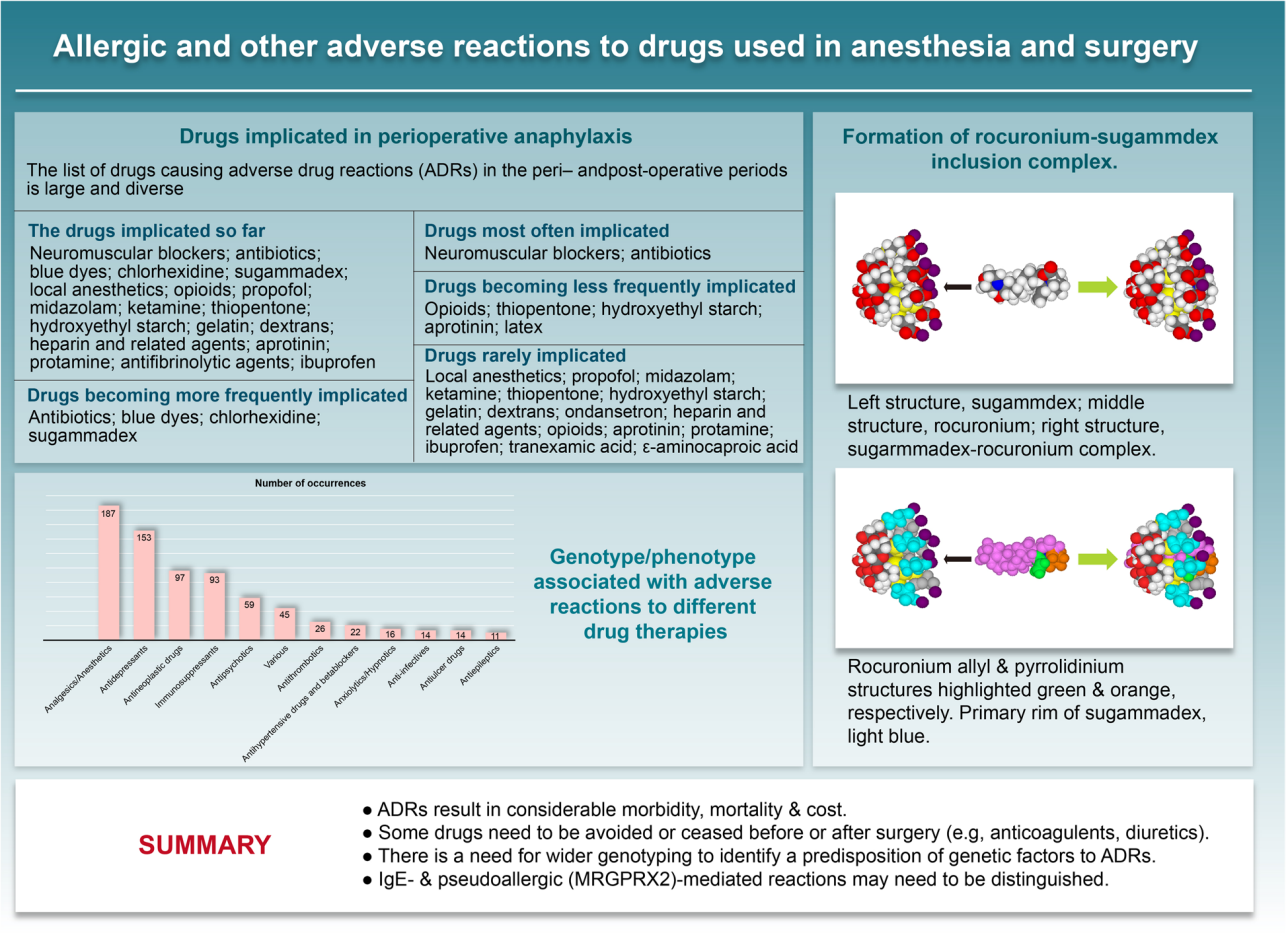

## Introduction

Considering the collective number of possible different drugs being taken by patients for existing conditions before anesthesia and surgery [1], the variety of drugs used during the perioperative period [2, 3], and range of medications administered postoperatively, especially for pain [4, 5], it is apparent that anesthetists and surgeons need to be constantly aware of the possibility of adverse drug reactions (ADRs) that may affect patient recovery and management. ADRs account for a significant number of hospital admissions and result in considerable morbidity, mortality, and cost. One UK study, for example, found an admission prevalence of 6.5% [6] while the incidence of serious ADRs in US hospitals was 6.7% with a fatality rate of 0.32% [7].

The extensive collective list of drugs used in anesthesia and surgery in the perioperative period includes induction agents (propofol, midazolam, ketamine, and possibly thiopentone); neuromuscular blocking drugs (NMBDs); opioids; antibiotics, particularly penicillins and cephalosporins; sugammadex; colloids; local anesthetics; polypeptides such as protamine; antifibrinolytic agents; heparin and related anticoagulants; blue dyes for sentinel lymph node localization; chlorhexidine; and others ([8]; see below for information on the individual background drugs in this list). In considering adverse reactions for an individual patient at any one time, one must include the total list of medications the patient is currently or has recently been exposed to. That will be the sum of drugs routinely, or occasionally, being taken (including over-the-counter products) for existing conditions, plus drugs administered preoperatively and postoperatively. Drugs administered postoperatively will, of course, be subject to length of patient recovery periods and ongoing treatments so the collective list of drugs for many different patients with different clinical conditions will be diverse and extensive.

Here we examine some properties, and interactions associated with drug-induced adverse effects together with genetic and non-genetic factors influencing enzyme function and drug metabolism and where ongoing research based on patient genotyping promises to better explain patient responses to a wide range of treatments. This is particularly so for patient responses to analgesic treatments employed in anesthesia and surgery. This approach, together with detailed information of a patient’s current medication and histories of previous drug-induced reactions; clinical descriptions of reactions to individual drugs; diagnostic details; and, where known, underlying mechanistic insights; provides the physician with the best combination of information to anticipate a potential ADR and be already prepared to deal with it.

## Anticipation of potential adverse drug effects

There are some potential culpable drugs that need to be considered before anesthesia and surgery. Examples include the claim that the use of diuretics in critically ill patients with acute renal failure is associated with an increased risk of death [9] and the possible association between preoperative antihypertensive management of medication and postoperative acute kidney injury after major vascular surgery. Relevant drugs include angiotensin-converting enzyme inhibitors, angiotensin II receptor binding inhibitors, calcium channel blockers, and diuretics. Note, however, that lack of consensus between guidelines on this subject remains [10, 11]. Anticoagulants that increase the chance of bleeding, for example, warfarin, enoxaparin, and P2Y12 inhibitors such as clopidogrel, prasugel, and ticlopidine (Sections 5.8 and 6) might also be ceased for a few days before elective surgery depending on the patient’s thrombotic risk [12].

In addition to known drug-induced hypersensitivities of some patients, including rare cases of anaphylaxis [6–8, 10], patients receiving a combination of two or more serotonergic drugs may carry the risk of serotonin toxicity [13]. Those patients already taking monoamine oxidase inhibitors (MAOIs), selective serotonin reuptake inhibitors (SSRIs), serotonin norepinephrine reuptake inhibitors (SNRIs), 3,4- methylenedioxymethamphetamine (MDMA or ‘ecstasy’), or tricyclic antidepressants are particularly at risk if some opioids, namely those known to be serotonin reuptake inhibitors (especially tramadol, meperidine, and perhaps tapentadol and fentanyl), are given during or soon after anesthesia [14].

## Genotyping and phenotyping to individualize drug therapy

Variations in the germline affect drug responses and such variants, and drug-drug interactions, are a major source of different individual drug responses. Genotyping and phenotyping tests are complementary approaches to individualize drug therapy [15] and an important tool in the study of ADRs [16, 17]. Early important findings from studies of the possible predisposition of genetic factors with ADRs found associations of human leukocyte antigen (HLA) genes with some serious delayed immune-mediated cutaneous reactions and liver injuries starting with the association of HLA-B*57:01 with abacavir hypersensitivity syndrome [17–19]. Table 1 lists some examples of HLA alleles associated with causative drugs and different hypersensitivity reactions including allopurinol-, carbamazepine-, and sulfamethoxazole-induced Stevens-Johnson syndrome (SJS) and toxic epidermal necrolysis (TEN); carbamazepine-induced maculopapular rash (MPR) and drug reaction with eosinophilia and systemic symptoms (DRESS); and amoxicillin-clavulanic acid with drug-induced liver injury (DILI) [20].

**Table 1:** Associations between severe hypersensitivity reactions and HLA alleles

**Causative Drug**	**Hypersensitivity reaction**	**HLA-alleles**	**Ethnicity**
Abacavir	Abacavir hypersensitivity syndrome	HLA-B*57:01	European descent, African-American, Hispanic descent
Allopurinol	SCAR	HLA-A*33:03 HLA-B*58:01 HLA-C*03:02	European descent, Han-Chinese
		HLA-B*58:01	Japanese, Thai
Amoxicillin-clavulanic acid	DILI	HLA-A*02:01 HLA-A*30:02 HLA-DQA1*01:02 HLA-DQB1*06:02 HLA-DRB1*15:01 HLA-DRB5*01:01	European descent
Carbamazepine	SJS/TEN DRESS	HLA-A*31:01 HLA-A*31:01 HLA-B*07:02	European descent
	MPR SJS/TEN	HLA-A*02:01 HLA-A*24:02 HLA-A*33:03 HLA-B*15:02 HLA-B*15:11 HLA-B*40:01 HLA-B*58:01 HLA-C*01:02 HLA-C*03:02 HLA-C*08:01 HLA-DQB1*03:03 HLA-DRB1*04:05 HLA-DRB1*07:01 HLA-DRB1*12:02	Han-Chinese
	DRESS	HLA-A*31:01 HLA-B*51:01	
	SJS/TEN	HLA-B*15:02	Indian
	SJS/TEN, MPR,EEM	HLA-A*02:06 HLA-A*31:01 HLA-B*51:01	Japanese
	SJS/TEN	HLA-B*15:11	
	SCAR	HLA-B*39:02	
	SCAR	HLA-A*31:01	Korean
	SJS/TEN	HLA-B*15:11	
	SJS/TEN	HLA-B*15:02	Malay, Thai, Vietnamese
	SJS/TEN, DRESS	HLA-B*46:01	Vietnamese
Sulfamethoxazole	SJS/TEN	HLA-B*38:01/:02/:11	European descent
	SJS/TEN	HLA-B*15:02 HLA-C*06:02 HLA-C*08:01	Thai

Reproduced from Brandt O, Bircher AJ. Delayed-type hypersensitivity to oral and parenteral drugs. J Germ Soc Dermatol. 2017;15:1111-32 [20] with permission from John Wiley & Sons

*SCAR* Severe cutaneous adverse reactions (AGEP, DRESS and SJS/TEN), *DILI* Drug-induced liver injury, *SJS/TEN* Stevenson-Johnson-syndrome/toxic epidermal necrolysis, *DRESS* Drug reaction with eosinophilia and systemic symptoms, *MPR* Maculopapular rash

**Fig. 1:**
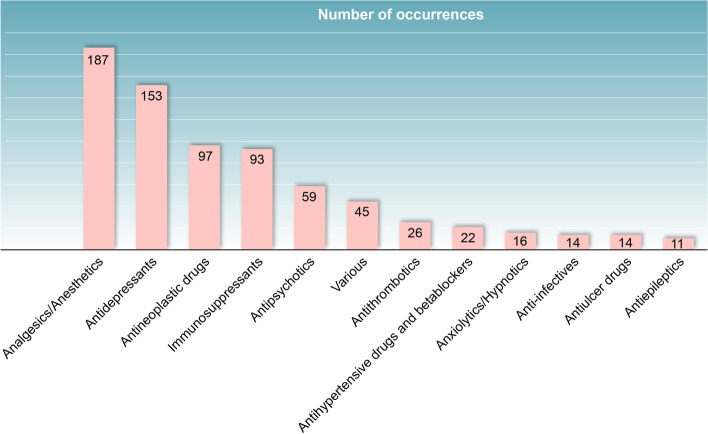
Genotype and/or phenotype associated with adverse drug reactions/high drug levels and different clinical conditions. (Reproduced and modified from Lorenzini KI, Desmeules J, Rollason V, et al. CYP450 genotype-phenotype concordance using the Geneva Micrococktail in a clinical setting. Front Pharmacol. 2021;12:730637. doi: 10.3389/fphar.2021.730637 [15], an open-access article distributed under the terms of the Creative Commons Attribution License (CC BY))

Cytochrome CYP450 enzyme genotyping and/or phenotyping for 537 patients (241 had genotyping and phenotyping, 61 genotyping only and 235 phenotyping only) were undertaken to test for inefficacies of drug treatment (i.e., low drug levels) and ADRs (reflected in high drug levels). Genotyping was found to correctly predict poor metabolizer phenotypes for most CYP isoenzymes but results for normal, intermediate, and ultra-rapid metabolizers were more variable. Figure 1 summarizes the associations between phenotype and/or genotype and clinical responses for different drug groups. Analgesic/anesthetic drugs, antidepressants, antineoplastics, and immunosuppressants showed the highest numbers of association occurrences. The genotype-phenotype results explained fully, or at least partly, 44% of the particular clinical event, the main ones being ADRs to analgesic/anesthetic drugs (*n* = 187), antidepressants (*n* = 153), antineoplastics (*n* = 97), and immunosuppressants (*n* = 93) [15].

Variations of CYP enzymes, the primary multi drug transporter P-glycoprotein (permeability glycoprotein, P-gp), and the enzyme catechol-*O*-methyltransferase that catabolizes catecholamines, have been used to assess ADRs and non-responses to drugs including analgesic drugs used for chronic pain, particularly opioids. Catechol-*O*-methyltransferase polymorphisms also affects opioid dose with the dose lower for wild-type compared to the mutated genotypes [16]. For the assessment of ADRs, CYP450 are the most studied enzymes. In a study to evaluate the link between lack of effective, or adverse drug therapy, patients’ genotype and/or phenotype were obtained to assess CYP and P-glycoprotein activities in pain clinic patients referred for an ADR or a non-response to chronic pain. The majority of patients studied involved an ADR (59.7%) while 37.9% were non-responders. The prodrug opioids (tramadol, codeine, oxycodone, dextromethorphan), were most involved followed by other opioids, antidepressants, nonsteroidal antiinflammatory drugs (NSAIDs), and acetaminophen (paracetamol). CYP and P-gp metabolic pathways for some analgesics are summarized in Table 2 [16]. Results showed a link between an ADR and modified CYP and P-glycoprotein in a high proportion of cases and this was especially apparent when the drug was a prodrug opioid, for example, tramadol and codeine, and its link with CYP2D6 and clinical outcomes (see below).

**Table 2:**
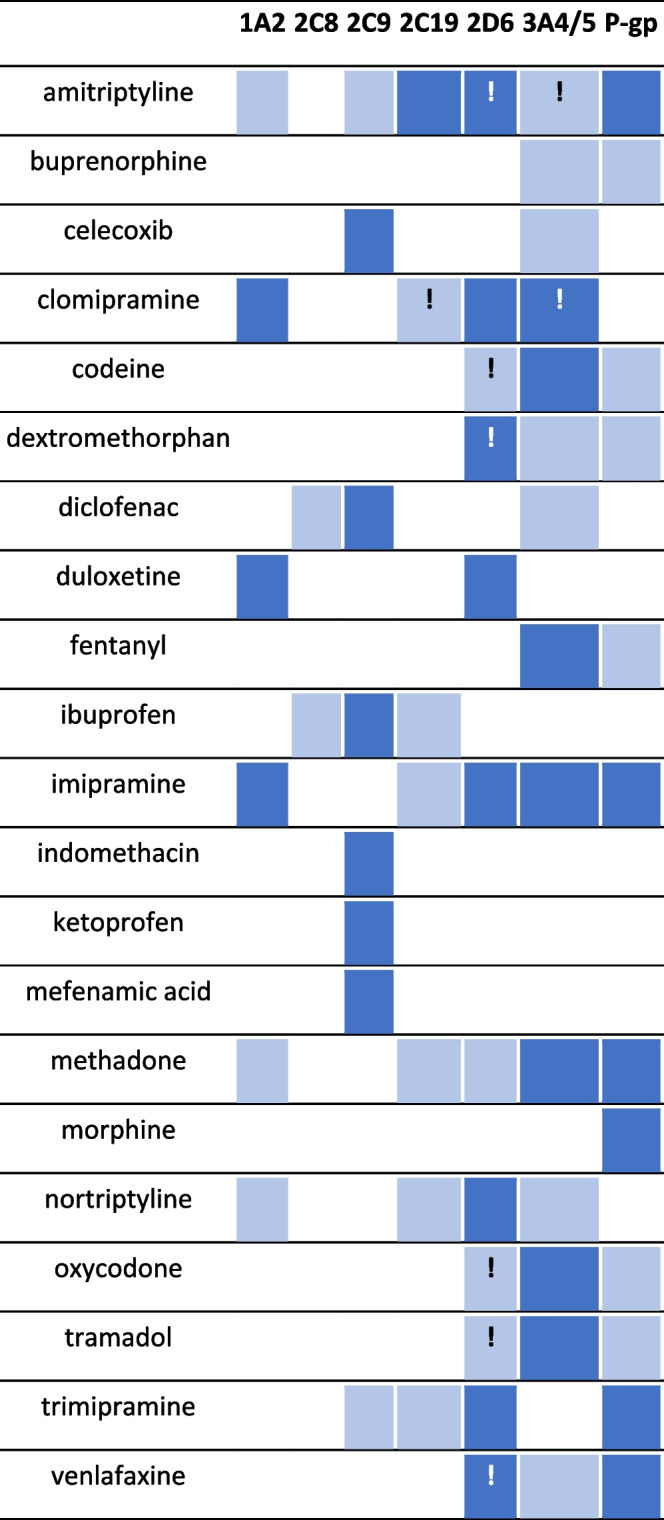
Analgesics and their cytochrome P450 (CYP) and P-glycoproten (P-gp) metabolic pathways

Major pathway  ; Minor pathway  ; Active metabolite

Reproduced from Rollason V, Lloret-Linares C, Lorenzini KI, et al. Evaluation of phenotypic and genotypic variations of drug metabolising enzymes and transporters in chronic pain patients facing adverse drug reactions or non-response to analgesics: A retrospective study. J Pers Med. 2020; 10:198. doi: 10.3390/jpm10040198 [16], an open access article distributed under the terms and conditions of the Creative Commons Attribution (CC BY) license (http://creativecommons.org/licenses/by/4.0/)

A European study, designated PREemptive Pharmacogenomic testing for Preventing Adverse drug REactions (PREPARE), has recently been established with the aim of examining individual germlines to guide optimal drug dosage and provide safer and more effective drug treatments. Described as an attempt to remove the “trial and error” approach to drug prescribing and progress toward personalized medication, the strategy involves more than 6,000 patients and the testing of a panel of 48 genetic variants in 13 pharmacogenes with the aim of reducing the incidence of ADRs to 39 target drugs [21, 22].

As mentioned above, genetic polymorphisms of patients, particularly CYP450 enzymes, e.g., genotypes CYP2C9, CYP2C19, and CYP2D6, which may affect drug detoxification, activation, and plasma concentration, may need to be considered as possible contributors to the individual variability of patient responses to some drugs (Table 2). With opioids for example, enzyme CYP2D6 converts the prodrugs tramadol to *O*-desmethyltramadol [23] and codeine to morphine [24, 25]. *O*-Desmethyltramadol is six times more potent as an analgesic than the parent drug while morphine shows a 200-fold greater affinity for the μ receptor than codeine. However, different allelic variants of CYP2D6 genotype produce different enzyme functions: CYP2D6*1 and CYP2D6*2 show normal function; for CYP2D6*9 and CYP2D6*10 function is decreased; with CYP2D6*3 and CYP2D6*4 function is absent; and a duplication of alleles such as CYP2D6*1, CYP2D6*2, and CYP2D6*35 leads to increased function. With tramadol for example, consequences of such allelic variants can therefore result in a range of effects from lesser pain relief for patients with no, or decreased, CYP2D6 activity (resulting in less tramadol converted to *O*-desmethyltramadol) to a life-threatening outcome in patients who are ultrarapid metabolizers and who therefore require more drug for pain relief [25–27].

The above brief examples serve to illustrate some of the sort of potential adverse reactions that might be anticipated by careful consideration of a patient’s clinical history, list of current medications, germline variations shown by genotyping and phenotyping, and in some cases, comparative epidemiology concerning the risk of different drug reactions between different countries [28]. Beyond that, however, the rareness and apparent random nature of drug-induced adverse effects, both immune- and non-immune-mediated, makes predictions difficult, meaning that there is no substitute for vigilance in the assessment of each patient and, at the very least, having some knowledge of what is already known of adverse reactions to the relevant drug(s).

Drugs commonly used in anesthesia and surgery together with reports of their involvements in allergic or other adverse reactions are now examined.

## Perioperative drug reactions: clinical features, comparative epidemiology, surveys, and incidences of drug groups involved

Although anesthesia today has an impressive record of safety, small but significant risks that are hard to overcome remain. These risks include an adverse response to the rapid administration of a variety of potent drugs over a short period of time, patients cannot draw attention to early symptoms, drapes can cover cutaneous symptoms such as pruritus and rash, some already sick patients may show abnormal responses, and there may be an early absence of symptoms in a condition showing only a single manifestation. A pseudoallergic, or anaphylactoid, reaction, for example, a drug-induced release of histamine and perhaps other allergic/inflammatory mediators, may show the same clinical picture as a true immune-mediated anaphylactic response making it difficult to distinguish the two. In such cases, identification of the mechanism of the reaction is important for subsequent patient safety during drug treatments and anesthesia [8].

Symptoms of anaphylaxis and anaphylactoid responses are often similar, making it difficult to distinguish the two based on symptoms alone although the former reaction tends to be more severe [8]. On a graded scale, most anaphylactic reactions are classified as grade 2 or 3 (and sometimes 4) depending on presence of cutaneous symptoms. Mild reactions are grade 1, moderate reactions grades 2–3 and severe reactions grade 3–4 [29–31]. In drug-induced reactions during the perioperative period, the full list of possible symptoms does not always manifest in every patient [32–36]. Cardiovascular collapse, the sole feature in ~60% of cases, is due to vasodilation and pooling of peripheral blood which reduces venous return and cardiac output. It is the most common, and usually the worst, life-threatening feature. It may not be the only sign, for example, asthmatic patients may experience bronchospasm which can be the first indication of a reaction and persisting lung inflation is often a difficult problem to reverse. Bronchospasm, more often seen in anaphylaxis, and the sole feature in up to about 20% of cases, may be critical since the high pressures needed for inflation reduce venous return and may increase ventricular compliance. It is not clear if the human heart is a target organ in anaphylaxis [37]; cardiac failure may occur in patients with cardiac disease during anaphylaxis but not in patients with normal cardiac function. Non-cardiogenic pulmonary edema is occasionally seen as a single feature of anaphylaxis and a postmortem finding, for example, due to reaction to protamine after cardiac bypass surgery. Angioedema or laryngeal edema may progress slowly making it prudent to prolong patient observation. A list of gastrointestinal symptoms which may last up to 6 h, includes abdominal pain, vomiting, diarrhea, and hematemesis. Cutaneous symptoms are more often seen in anaphylactoid (non-IgE-mediated) reactions. Other possible signs and symptoms can be grouped as: Uncommon features — cardiac failure, disseminated intravascular coagulation, hemoptysis, melena. Minor features — rash, flushing, rhinitis, cough, lacrimation, urticaria, pruritus, aura, conjunctivitis. Late features — headache, edema, thromboembolism, wound hematoma, vaginal discharge [32–35].

Given the enormous number of anesthetics administered throughout the world, for example recent estimates of 45 million and 8 million per year in the USA and France, respectively, drug-induced immediate hypersensitivity or type I IgE-mediated reactions and anaphylactoid reactions during the perioperative period are rare. Early reports on anaphylaxis in anesthesia date to Fisher and More in 1981 [38] who found an incidence of 1 in 5,000 to 1 in 25,000 with a mortality rate of 3.4%. In a later, more comprehensive series, Fisher and Baldo [33] estimated an incidence of 1 in 10,000 to 1 in 20,000. Subsequent estimates from different countries generally show estimated incidences ranging from ~1 in 1,000–2,000 to 1 in 20,000. For example: Australia 1–5,000–20,000 and 1–11,000, New Zealand 1 in 1–5,000–13,000, U.K. 1 in 5,000–10,000, France 1–3,500–6,000 and 1–4,600–13,000; Spain 1 in 10,200, Norway 1–6,000, Japan 1–10,000 and 1–18,600, Singapore 1 in 10,000, and 1–7000, and Thailand 1 in 5,000 [8].

The most comprehensive data on perioperative drug-induced anaphylaxis is contained in the Australian 30-year series maintained by Malcolm Fisher at Royal North Shore Hospital of Sydney, in the ongoing French series beginning with results from 1984–1989 and still underway, and the smaller 2005 Norwegian study [8, 33, 35, 36, 39–43]. Table 3 shows a side-by-side comparison of results from 606 patients in the Australian series and 1816 patients in France at that time. A summary of the most important features in these results includes the predominance of NMBD-induced reactions in both series; a high incidences of reactions to succinylcholine and rocuronium amongst the NMBDs; a much lower incidence of latex anaphylaxis occurred in Australia; penicillins and cephalosporins were the dominant culprit antibiotics with the former implicated more often in France and cephalosporins nearly five times more involved in the Australian reactions; opioids were implicated in only about 2% of reactions; and gelatin proved to be the predominant colloid in both series. For more than three decades French investigators have published a total of 11 surveys covering the years 1984–2020. During that time, NMBDs have remained the predominant cause of anaphylaxis (see Section 5.2), reactions to induction agents and opioids have decreased (Sections 5.1 and 5.8), drugs such as colloids (Section 5.7) have remained stable but marked increases in reactions to antibiotics (Section 5.4) and blue dyes (Section 5.6) have occurred.

**Table 3:** Agents responsible for type I immediate allergic reactions during anesthesia [8]

Agent	**Reaction (%)** **France**^**a**^	**Reaction (%)** **Australia**^**b**^
**Neuromuscular blocking drugs**	**58.1**	**61.9**
Succinylcholine	33.4	32.8
Rocuronium	29.3	16.8
Atracurium	19.3	9.1
Vecuronium	10.2	5.6
Pancuronium	3.6	1.9
Mivacurium	2.5	0.5
Cisatracurium	1.7	0.5
Alcuronium^c^		24.8
*d*-Tubocurarine		2.9
Gallamine		2.1
More than one drug^d^		2.1
**Hypnotics/Induction agents**	**2.3**	**10.4**
Propofol	55.8	6.3
Midazolam	32.6	
Thiopentone	9.3	52.4
Ketamine	2.3	
Alfathesin		30.2
Propanidid		9.5
Methohexitone		1.6
**Latex**	**19.7**	**0.8**
**Antibiotics**	**12.9**	**8.6**
Penicillins	49.0	15.4
Cephalosporins	37.0	73.1
Vancomycin		5.8
Others	14.0	5.8
**Colloids**	**3.4**	**4.6**
Gelatin	89.9	85.7
Hetastarch	9.5	
Albumin	1.6	
Dextran 70		14.3
**Opioids**	**1.7**	**2.6**
Morphine	35.5	50.0
Fentanyl	22.6	25.0
Sufentanil	22.6	
Nalbuphine	12.9	
Remifentanil	6.5	
Meperidine (Pethidine)		18.7
Omnopon		6.3
**Other agents**^**e,f**^	**2.7**^**e**^	**3.8**^**f**^
**No causal drug detected**		**7.4**

From Baldo BA, Pham NH [2021]. Drug allergy: clinical aspects, mechanisms, diagnosis, structure-activity relationships. 2^nd^ edition. Cham, Switzerland: Springer Nature. https://doi.org/10.1007/978-3-030-51740-3 [8]. Reproduced with permission from Springer Nature

^a^Survey in France 1997-2004; 1816 patients. Data from Mertes PM, Alla F, Trechot P, et al. Aaphylaxis during anesthesia in France: an 8-year national survey. J Allergy Clin Immunol. 2011;128:366–73 [35]

^b^On-going Australian survey; 606 patients. Data from Fisher MM, Jones K, Rose M. Follow-up after anaesthetic anaphylaxis. Acta Anaesthesiol Scand. 2011;55:99–103 [43]

^c^Discontinued

^d^Eight reactions with two different neuromuscular blocking drugs administered

^e^Made up largely of patent blue, propacetamol, local anesthetics, aprotinin and protamine

^f^Made up of largely of induction agent plus neuromuscular blocker (4 patients), protamine, local anesthetics, patent blue, chlorhexidine, contrast media and ondansetron

Except for a few investigations in the U.S. involving small numbers of patients, no comprehensive and ongoing studies of this subject have been published. For many years there was less interest in the United States where reactions were said to be rare although it may be that there was less interest in the subject or reactions were largely unrecognized or went unreported. Following the early French and Australian surveys, numerous epidemiological studies of perioperative anaphylaxis, many retrospective, have been published; a recent count identified 55. However, as well as the small numbers of patients examined in many countries, the retrospective nature of the studies, failures to clearly identify culprit drugs, and uncertainties in distinguishing immune-mediated from non-immune-mediated anaphylactoid reactions are, as pointed out by Harboe et al. [39], recurring criticisms. There are few, or no, accessible published findings with adequate numbers of patients and new information, from many countries, including, but not exclusively, Africa, many Asian and Arab countries, the Indian Subcontinent, Central and South America, much of Eastern Europe and Russia. In addition, and in common with the early Australian, French, Scandinavian, and recent UK investigations (see below), NMBDs and antibiotics have been identified as the most important culprit drugs.

In a survey covering the period 2005–2012, the first multi-center retrospective examination from the UK, 161 patients were investigated for anaphylaxis. Skin and IgE antibody tests identified 103 patients with an IgE-mediated drug-induced hypersensitivity, 61 (59.2%) of whom reacted to a NMBD, 13 patients (12.6%) reacted to an antibiotic, 9 (8.7%) to patent blue V, and 8 patients (7.8%) to chlorhexidine [44]. Other drugs previously rarely implicated in type I allergic reactions were ondansetron (5 patients), midazolam (2) and local anesthetics (2 patients). Following the French lead, the recent UK 6th National Audit Project (NAP6) survey of perioperative anaphylaxis reviewed 266 reports of anaphylaxis grades 3–5 over a 1-year period from all UK 6th National Audit Project national health service (NHS) hospitals [45]. In 192 cases, the leading cause of reactions was antibiotics with 94 reactions, NMBDs were implicated in 65 reactions, chlorhexidine in 18, and patent blue in 9 with gelatin, ondansetron, sugammadex, propofol, protamine, and ibuprofen making up a total of 13 reactions. Opioids, still widely used but known to be rarely allergenic, were not involved in any of the cases. Approximate incidences of the most prevalent culprit drugs were patent blue 1 in 7,000, NMBDs 1 in 19,000, antibiotics 1 in 27,000, and chlorhexidine 1 in 127,500. Incidence of anaphylaxis per 100,000 for the two most implicated drug groups were NMBDs 5.3, antibiotics 4.

Overall then, results from a relatively small number of clinically adequate surveys from different countries (see expanded review in [8]), reveal that NMBDs and antibiotics provoke a clear majority of perioperative allergic reactions. More detailed analyses of the involvement of the different drug groups are shown in the relevant sections below.

## Individual drug groups implicated in reactions

Adverse drug reactions during the postoperative period also need to be anticipated since patients, both those remaining in hospital or discharged, may remain on, or commence, new therapies. These may include NSAIDs and opioids for pain relief; a variety of anti-clotting agents (e.g., the vitamin K antagonist warfarin, antiplatelet drugs, direct oral anticoagulants, heparin including low molecular weight forms, synthetic pentasaccharide inhibitors of factor X_a_, and direct thrombin inhibitors), proton pump inhibitors to treat gastroesophageal reflux disease; and a variety of other therapies for ongoing blood pressure, heart, vasculature, lung, kidney, and other diseases.

In the following sections, the drugs shown to be the leading causes of anaphylaxis during anesthesia in the most comprehensive surveys from France and Australia and more recent surveys involving lesser patient numbers, are discussed in detail. In addition, a list of other drugs, less often involved in provoking reactions, but none the less important, are summarized.

### Hypnotics/induction agents

Before being replaced by propofol and, to lesser extent etomidate, Cremophor-based induction agents alfathesin and propanidid contributed significantly to life-threatening anaphylaxis during anesthesia with incidences as high as 1 in 875 cases. This was clearly shown in an early Australian survey involving 443 patients where thiopentone, alfathesin, and propanidid accounted for 52.6%, 37.2% and 7.7%, respectively of anaphylactic reactions to hypnotics [33]. As well as propofol, the main induction agents now widely used are midazolam and ketamine with thiopentone only occasionally used in, for example, some cases involving electroconvulsive therapy.

#### Thiopentone

Thiopentone can be viewed as the classic drug used in anesthesia for rapid sequence induction. Since its introduction in 1934 and heavy usage over many years, reports of its involvement in hypersensitivity reactions are rare although it seems likely that many cases may have been unrecognized or misdiagnosed. Incidences of anaphylaxis were stated to be 1 in 22,000–29,000 [46, 47]. In 1985 Boileau et al. [48] in France reported 258 cases of anaphylaxis to the induction agent while the early Australian and French surveys of 606 and 1816 patients, respectively found incidences of immediate allergic reactions of 5.4% and 0.2%, respectively (Table 3). The majority of reactions occur after multiple exposure to the drug and although a few cases of anaphylaxis have been reported after one or two exposures [8, 49], including one case after a 20-year gap in exposure [50], it is generally believed that at least six exposures are usually required. Reactions to thiopentone sometimes include cutaneous symptoms of rash, urticaria and severe exfoliative dermatitis. Reactors to the drug tend to be older with a female to male ratio of 3:1.

For the diagnosis of immediate allergic reactions to thiopentone, challenge tests were used, sometimes with adverse consequences, leading to preferment of skin testing. Prick testing is carried out with undiluted solution (25 mg/ml). Intradermal testing starts with a dilution of 1 in 10,000 and proceeds up to a maximum of 1 in 10 (2.5 mg/ml). Serum IgE antibody tests together with inhibition studies to ensure specificity of binding [51, 52] and controls for false positives due to high levels of IgE antibodies to substituted ammonium groups [53–55] proved a valuable test in helping to confirm immediate allergic reactions to the hypnotic [52, 54, 56, 57]. Employment of the thiopentone IgE immunoassay together with selected barbiturate structural analogs in quantitative inhibition studies enabled the identification of the IgE antibody binding structures on the thiopentone molecule. These proved to be position 1 on the pyrimidine ring with its attached sulfur atom and, on the other side of the ring, the ethyl and secondary pentyl groups at position 5 [54, 55].

#### Propofol

Propofol (Diprivan^®^), used for short-acting induction and maintenance of anesthesia, and in intensive care and outpatients for short procedures, is an oil-in-water emulsion formulated with soybean oil and egg phospholipid as emulgent. Two other formulations, the microemulsion Aquafol^®^ and water-soluble phosphate derivative prodrug Lusedra^®^ which is metabolized to propofol, have been introduced. Apart from several isolated case reports ([58] and references therein], propofol is regarded as a remarkably safe drug with a reported incidence of 1 in 60,000 for allergic reactions [59]. French and Australian surveys have shown low incidences of anaphylaxis of 1.3% and 0.65%, respectively during anesthesia (Table 3).

Although there are a small number of reports of suspected allergy to propofol in individuals allergic to egg, soy or peanut, the first in 1994 [60], convincing confirmatory evidence has been absent. This, and some other reports of possible allergies after propofol in children with food allergy led to the conclusion that egg allergy might be a possible risk for the drug’s administration. However, several investigations have not found evidence to support this. In a study of 60 patients with eosinophilic esophagitis who received propofol, 52 (87%) of whom were sensitized to egg, soy, or peanut, no allergic symptoms were seen [58]. Of 153 patients allergic to egg, soy, or peanut and exposed to propofol, skin and challenge testing revealed 4 with allergy to propofol but none had symptoms of allergy or IgE antibodies to egg, soy, or peanut. In an extension of the study, no cases of propofol allergy were identified in anesthetic charts of 99 patients with IgE antibodies to egg, soy, or peanut [61]. More recent investigations also found no evidence of a relationship between food allergy history and perioperative reactions to propofol [62], that allergic reactions to propofol are rare, and they are not reliably predicted by a history of food allergy [63].

Overall, it seems that in cases of propofol hypersensitivity, the propofol molecule itself is the source of the allergic sensitization. This conclusion gains support from the report of anaphylactic reaction to Aquafol^®^ which is formulated without the lipid solvent in propofol emulsion [64].

For skin testing, the prick test concentration is 10 mg/ml while for intradermal application, testing starts at a dilution of 1 in 100–1,000 proceeding up to a 1 in 10 dilution (1 mg/ml).

#### Midazolam

Midazolam (Versed^®^), a benzodiazepine, is a short-acting (half-life 1.8–6.4 h), rapidly effective central nervous system (CNS) depressant when given intravenously. Indicated for procedural sedation, and often given with fentanyl, midazolam acts by binding to the gamma-aminobutyric acid (GABA) receptor complex producing hypnotic, sedative, amnestic, muscle relaxant, and anticonvulsant actions. It is also administered intramuscularly, orally, rectally, and intranasally.

Hypersensitivity reactions to midazolam are uncommon although a retrospective skin and provocation tests study from Brazil covering 101 patients over a 10-year period concluded that the drug is a major cause of intraoperative immediate hypersensitivity [65]. Surprisingly, 10 of 28 patients (35.7%) tested positive, a frequency similar to that found for NMBDs (22 of 62; 35.5%). A comparison with results in the early Australian and French surveys of 606 and 1816 patients, respectively showed reactions to NMBDs in 58–60% of allergic patients and to midazolam in 0.75% of French patients and no reactions in the Australian survey (Table 3). Allergic reactions to the hypnotic have been described after intranasal [66, 67] and intrarectal [68] administration. There are at least 7 reports of anaphylactic/anaphylactoid reactions to midazolam [8, 69–72] although confirmatory diagnostic tests were not always undertaken. Numerous reports of urticaria and rash in patients receiving midazolam, including some cases of anaphylaxis, have led to the suggestion that there may be an association between midazolam-induced anaphylaxis and allergic urticaria and special attention should therefore be paid to this [71]. A clear correlation between the two, however, has not been established. Other suspected hypersensitivities and non-immune-mediated adverse reactions recorded for midazolam include dyspnea, pruritus, laryngospasm, respiratory depression, tonic clonic seizures, cardiac arrhythmias, facial edema, and eyelid swelling, [72, 73].

Recommended skin test concentrations for midazolam show some variations: perioperative anaphylaxis investigation guidelines issued by the Australian and New Zealand Anaesthetic Allergy Group (ANZAAG) [74] recommend 1 mg/ml for prick testing and 10 µg/ml (initial) up to 100 µg/ml for intradermal testing while the stated maximum non-irritant concentration s 0.5 mg/ml. A 2019 European Network on Drug Allergy (ENDA)/European Academy of Allergy and Clinical Immunology (EAACI) position paper [75] for drugs used perioperatively suggests 50 µg/ml for intradermal testing. Some other European references recommend a prick test concentration of 5 mg/ml and up to a maximum of 0.5 mg/ml for intradermal testing.

#### Ketamine

Ketamine (Ketalar^®^) is used for a number of purposes including the initiation and maintenance of anesthesia; for procedural sedation; as a sedative in emergency departments and intensive care; for acute and chronic pain; as an induction agent for pediatric patients; in the dental surgery; and as a rapidly acting antidepressant. Most preparations are racemic mixtures, composed of dextrorotatory *S*-( +)-ketamine (Ketanest^®^ and Ketanest-S^®^) and the less active enantiomer *R*-(−)-ketamine. Both enantiomers bind to the *N*-methyl-*D*-aspartate (NMDA) receptor, the *S-*enantiomer doing so with three-times greater affinity. Binding also occurs with other binding sites including opioid, nicotinic, muscarinic, and some ion channels.

Ketamine induces histamine release from skin and lung mast cells [76] which may account for some reported anaphylactoid and unusual reactions [77–80] but immune-mediated anaphylactic reactions, although rare, have been reported. Two such true immediate type I reactions include a case following ketamine infusion confirmed by positive ketamine skin tests and elevated serum tryptase levels [81] and a case diagnosed as a grade IV anaphylaxis (World Allergy Organization grading system) with positive skin tests and elevated serum tryptase and histamine levels [82]. Perhaps the most feared adverse reaction to ketamine is laryngospasm seen, for example when given to a delirious patient [83] and in a patient requiring adrenaline where it occurred together with a generalized rash [84].

There are several reports of adverse reactions to ketamine in children. A 2009 analysis of 8,282 patients’ data found that ketamine risk factors for airway and respiratory adverse events are high intravenous doses of the drug, administration to children < 2 years or ≥ 13 years old, and coadministration of anticholinergics or benzodiazepines [85]. Reports of reactions in children include details of a 4-year old child who developed urticaria after intravenous midazolam and was diagnosed as hypersensitive to both ketamine and midazolam after proving intradermal test-positive to ketamine at 1 mg/ml [86]; a 6-year old with wheeze and widespread urticaria disseminating from the intramuscular ketamine injection site [87]; and a 9-year old who showed facial edema, erythema on the neck, and labored breathing after 30 mg intravenous ketamine. This patient proved negative to a skin prick test concentration of 1 mg/ml but positive to intradermal tests at concentrations of 1 and 0.25 mg/ml [88].

Overall though, ketamine is deemed to be a relatively safe and effective choice for procedural sedation in children.

Recommended concentrations of ketamine solutions for skin testing show a wide variation depending on the particular issued guidelines. For example, the British Society of Allergy and Clinical Immunology [89] suggest 10 and 1 mg/ml for prick testing and up to a maximum of 1 mg/ml for intradermal testing while both ANZAAG [74] EAACI guidelines, 2019 [75] recommend 100 mg/ml for prick testing and up to 100 µg/ml for intradermal testing.

### Neuromuscular blocking drugs (NMBDs)

A decade after the introduction of muscle relaxants into anesthesia [90], Foldes et al. [91] declared: “…[the] first use of muscle relaxants in anesthesiology by Griffith and Johnson in 1942 not only revolutionized the practice of anesthesia but also started the modern era of surgery and made possible the explosive development of cardiothoracic, neurological and organ transplant surgery.”

As outlined above, since the early surveys of drug-induced anaphylactic reactions in the perioperative period (Table 3), NMBDs have been found to be the drugs implicated most often. In the consecutive French series, the incidences of reactions to NMBDs range from 81% in the 1984–1989 survey to a low of 48% percent in the 2005–2007 survey (average of all surveys 61.2%). The selection of NMBDs used over the last 30 years has changed, nevertheless, usage of succinylcholine has remained relatively high as has the numbers of cases of anaphylaxis to the drug. Anaphylaxis is more common with succinylcholine and rocuronium than with atracurium and rocuronium appears to be of higher risk compared to pancuronium, vecuronium, and cisatracurium [44, 44, 92, 93]. In the UK NAP6 survey, NMBDs were judged responsible for 34% of definite/probable cases of anaphylaxis, causing 32% of deaths or cardiac arrests. Rocuronium was implicated in 42% of reactions to NMBDs, while atracurium and succinylcholine were involved in 35% and 22% of cases, respectively [45]. Of 83 cases of anaphylaxis during anesthesia detected in a 1996–2001 Norwegian study, 71% proved to be IgE antibody-mediated, 93.2% of these were mediated by NMBDs, and succinylcholine was the NMBD most often implicated followed by rocuronium and vecuronium [39].

The ratio of females to males for allergy to NMBDs is up to about 4:1, atopy is not a risk factor, and the median annual incidences of allergic reactions to NMBDs has been estimated to be 105.5, 250.9, and 184 per million procedures for men, women, and children (both sexes), respectively. Peak ages for anaphylaxis are 10–20 and 40–60 years for males and 40–50 years in the high incidence range of 30–60 years for women [33, 35].

Early skin testing with free NMBDs in the late 1970s and 1980s [94–98] to diagnose what appeared to be anaphylactic reactions to the drugs had become the standard diagnostic procedure by the 1990s [99, 100]. This was soon supplemented by immunoassays demonstrating the involvement of long persisting IgE antibodies [101] that recognized, and cross-reacted with, quaternary and tertiary ammonium groups on the different NMBD molecules [102–106]. Experiments also demonstrated cross-reactivity with substituted ammonium ions on a range of different chemicals and drugs with diverse pharmacological activities, suggesting prior immune sensitization to ammonium ions in the NMBD-allergic patients who reacted to an NMBD on first exposure [103]. Subsequent studies tend to support this explanation but it remains unexplained why only a very small number of subjects with IgE antibodies to ammonium groups demonstrate allergic sensitivity to NMBDs [107]. Routine diagnosis of allergic type I sensitivity to a NMBD is now undertaken by a combination of skin testing with the free drug(s) (Table 4), NMBD-specific IgE immunoassays (if available), otherwise with a diagnostically useful cross-reacting morphine immunoassay prepared in-house or as a commercial product [108, 109], a basophil activation test [110], and tryptase testing [111]. The simplicity of the morphine-solid phase assay and its suitability for routine laboratory use makes it a valuable addition to skin testing in diagnosing NMBD allergic sensitivity. Failure of the morphine-based immunoassay to detect IgE antibodies to the tetrahydroisoquinolinium NMBD atracurium in a significant number of patients [108, 109] raises the question of the specificity of the atracurium-reactive IgE, in particular, whether the antibodies are complementary to substituted ammonium groups. Employment of an atracurium solid phase and inhibition studies demonstrated specific IgE binding inhibited by atracurium but not by six other NMBDs [112]. Interestingly, the patients with reactive IgE antibodies each had a history of more than 20 operations, suggesting prior sensitization.

**Table 4:** Concentrations^a^ of neuromuscular blocking drugs used for skin testing

**Neuromuscular blocking drug**	**Skin prick test**^**b**^** concentration mg/ml**	**Intradermal test**^**c**^** concentration µg/ml**
Succinylcholine	10	100
Rocuronium^d^	10	50
Vecuronium^d^	4	40^e^
Pancuronium	2	20^e^
Atracurium	1	10
Cisatracurium	2	20^c^
Mivacurium	0.2	2

Positive control for prick test: Histamine 10 mg/ml or codeine phosphate 9% w/v. Negative control for prick and intradermal tests: Same volume of solvent used for drugs

^a^Maximum nonirritative concentrations normally non-reactive in subjects not allergic to a neuromuscular blocking drug

^b^A positive test is a wheal after 20 min with a diameter 3 mm greater than the negative control or a diameter at least half the diameter of the positive control

^c^0 02 – 0.05 ml injected to give a 4 mm diameter bleb. A positive test is the appearance of an erythematous wheal (often pruritic) after 20 min with a diameter at least twice that of the initial bleb

^d^A high proportion of positive reactions in normal controls has led to suggestions that these prick test concentrations are too high

^e^Some published maximums for vecuronium and pancuronium are 400 μ g and 200 μg, respectively. See also, [74, 75]

### Sugammadex

**Fig. 2:**
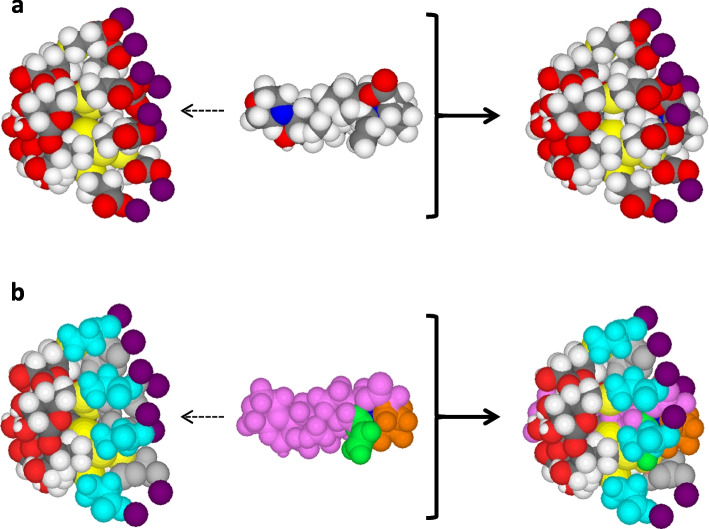
Diagrammatic representation of molecular models of sugammadex (left hand structures), rocuronium (middle structures), and rocuronium-sugammadex host-guest inclusion complex (right hand structures), showing the encapsulation of the neuromuscular blocking drug (NMBD) by sugammadex. **a** Formation of the inclusion complex shown in conventional colors for atoms. **b** Coloring of atoms changed to distinguish the rocuronium and sugammadex structures. Allyl group of rocuronium colored green; pyrollidinium group, brown; rest of rocuronium molecule, mauve. Four of the eight groups that make up the primary ring of sugammadex and visible on one side of the molecule, are shown in light blue. Conventional atom colors shown are H white, C black, O red, N blue, S yellow, Na violet. Adapted from Baldo BA and Pham NH (2021). Drug allergy: clinical aspects, diagnosis, mechanisms, structure-activity relationships, 2^nd^ edition. Springer, New York, p. 368. Reproduced with permission from Springer Nature

In seeking a method to aid solubility of the widely used NMBD rocuronium and decrease injection pain, a strategy was devised to encapsulate the rocuronium molecule to form an inclusion complex with a chemically modified γ-cyclodextrin, named sugammadex [113, 114] (Fig. 2). The high affinity and specificity of sugammadex for rocuronium (and other aminosteroid NMBDs) enabled its use in anesthesia for rapid reversal of rocuronium-induced neuromuscular block by sequestering the drug as an inclusion complex and removing it from the neuromuscular junction [113–116]. Despite successful encapsulation, doubt remains whether the ammonium ion at position 16 on the steroid nucleus of encapsulated rocuronium is completely enclosed by thio(2-carboxyethyl) sodium side chain groups at the primary ring of sugammadex or if it might still be accessible for binding with complementary IgE molecules [117]. This question is also relevant to the rocuronium tertiary ammonium group at the opposite end, the secondary rim, of the inclusion complex [115, 118].

The increasing use of sugammadex has been accompanied by a small but steady increase of reports of anaphylaxis/anaphylactoid reactions to the agent [119, 120]. This has been most apparent in Japan where sugammadex was first used in 2010 and where it has perhaps been used more intensively than elsewhere. An investigation of the drugs most often involved in anaphylaxis in Japan showed that, for the period 2012–2016, sugammadex with 32% of the cases was the biggest cause followed by rocuronium (27%) and antibiotics (23%) [121]. A Japanese retrospective study of 15,479 patients who received sugammadex revealed 6 cases of anaphylaxis (0.039%; 1 in 2,580) [122], an incidence similar to that for succinylcholine and rocuronium [91]. The incidence of rare adverse events is difficult to measure but the recent estimate of the incidence of anaphylaxis to sugammadex obtained in a retrospective single-center study over 3 years (2016–19) [123] is strikingly less than the Japanese estimate and the drug’s package insert figure of 1 in 300 [124]. In accounting for the difference, the not entirely convincing suggestion of regional differences in exposure to cyclodextrins was advanced.

The possibility of altered allergenic recognition — allergenicity enhanced, lessened, or abolished — with special reference to the rocuronium-sugammadex inclusion complex (S-R-Cx), suggested in 2011 [115] and subsequently shown to occur [125–131], raised the question of the basis of the observed altered immune recognition [132]. In a recent study of a patient who experienced IgE/FcεRI-dependent anaphylaxis to S-R-Cx, the patient’s serum, skin testing and the basophil activation test (BAT), were employed together with a panel of carefully selected structural analogs of rocuronium. Results showed that recognition of S-R-Cx is due to IgE interaction with a new allergenic determinant formed by a shape alteration of the attached thiocarboxyethyl sodium side chains at the primary ring of the host sugammadex molecule [131] (Fig. 2). It is clear that when an allergic reaction to sugammadex is suspected, skin, BAT and other testing should be undertaken with free sugammadex, rocuronium, and the complex, S-R-Cx, the latter prepared using the stoichiometric ratios of sugammadex and rocuronium [132].

The successful sequestration of rocuronium into an inclusion complex with sugammadex prompted the early suggestion that the modified γ-cyclodextrin might be a new and useful treatment to manage rocuronium-induced anaphylaxis [117]. Soon after, several reports were published [133–135] that appeared to support this speculation and there are now reports of at least 23 cases presenting data claiming, to at least some extent, mitigation of rocuronium-induced anaphylaxis and successful management after administration of sugammadex ([8, 119]; for full list contact author). Claims for non-IgE-dependent pseudoallergic reactions to rocuronium reversed by sugammadex have been made for three patients showing irritant skin reactions but no evidence of immediate hypersensitivity to the NMBD. In a confusingly presented report, “anaphylaxis” and elevated tryptase levels are described and attributed to mastocyte-related G-protein-coupled receptor (GPCR) member X2 (MRGPRX2)-mediated “pseudoallergic reactions” [136]. MRGPRX2, a low affinity, high-dose human mast cell receptor mediating nonimmune adverse reactions results in some pseudoallergies manifesting as itch, inflammation, and pain (Section 6). It is not yet clear whether rocuronium mediates mast cell activation and subsequent cutaneous reactions via activation of MRGPRX2 [137, 138] but if it does, sugammadex may suppress such reactions. However, of the claims so far of rocuronium-induced IgE/FcεRI-mediated anaphylaxis mitigated by sugammadex, symptoms and diagnoses overwhelmingly indicate systemic anaphylaxis with cutaneous reactions uncommon.

The effect of rapid reversal of anaphylactic symptoms is, of course, surprising since it is difficult to see how sugammadex could rapidly alleviate anaphylactic symptoms by stopping or reversing the rocuronium-induced allergic mediator cascade since that would seem to require sequestration of not only free rocuronium and perhaps IgE-bound rocuronium in plasma but also rocuronium complexed to IgE antibody at the FcεRI receptor on mast cells [139–141]. Other explanations have been advanced to explain the apparent improvement in some patients’ hemodynamic state after sugammadex. These include improvement due to already administered epinephrine and fluid resuscitation and the suggestion that more sugammadex sequesters rocuronium preventing further release of mediators allowing epinephrine to work. Attention has also been drawn to a dramatic recovery in a similar clinical situation after 15–20 min of traditional treatment with epinephrine and steroids [142]. A contrasting case is a report of successful treatment with sugammadex of anaphylaxis to rocuronium in a patient whose only symptom was bronchospasm. Despite the absence of prior treatment with epinephrine, the patient experienced a dramatic recovery ~15 min after receiving sugammadex [143]. In what the authors called a “case-control” study, Platt et al., [144] found that only six of 13 cases of what was thought to be anaphylaxis to rocuronium improved after sugammadex. Of the six, only three were confirmed to be due to rocuronium, bringing into question the original diagnostic conclusions and data interpretations. The authors concluded that in the absence of an explanation of the sugammadex-IgE-rocuronium-based mechanism, sugammadex is not effective in reversing rocuronium anaphylaxis but effective in some cases of non-rocuronium anaphylaxis by reversing neuromuscular blockade which increases muscle tone compressing intramuscular and intra-abdominal vessels thus reducing venous capacitance, increasing venous return, and cardiac preload [134]. Attention was also drawn to potential ventilation complications when reversing neuromuscular blockade in a situation of high airway pressures and hypoxia.

Explanations suggested above obviously need close consideration and, if possible, further investigation, but there remains a conspicuous absence of any examination of the rapid and sometimes dramatic improvement in the clinical picture in the growing list of cases of rocuronium-induced anaphylaxis treated with sugammadex. Rapid reversal of symptoms, often complete, is reflected in the temporally related statements describing the response to sugammadex as “immediate, “almost instantaneous”, hemodynamic improvement “2 min later”, “45 s later” and so on [8, 119]. Two in vitro models have been presented as evidence against sugammadex-induced mitigation of rocuronium-induced anaphylaxis -- one is the reported failure of sugammadex to block CD63 expression after rocuronium-induced basophil activation ([145] and the second the failure of sugammadex to reverse the course of an established allergic reaction to rocuronium in the skin [146]. In the BAT experiments, already expressed CD63 may not reflect termination of mediator release; with the cutaneous model, degranulation of mast cells is a rapid process which, once initiated, leads to wheal and flare reactions as a result of capillary permeability and vasodilation, respectively. Histamine liberation and an increase in local blood flow begin within 2 min but histamine alone does not account for resultant wheal size. Already liberated mediators and a cutaneous reaction may not by be prevented or even diminished by sugammadex [147–149].

Accumulation or absence of convincing case reports over a long period or fresh insights leading to new investigative approaches may contribute to resolving the question of sugammadex’s capacity to mitigate rocuronium-induced anaphylaxis but only a controlled clinical trial in humans involving challenge studies (which is unlikely), will ultimately decide the issue. In the meantime, some in vitro approaches may provide important relevant data. Along with experimental strategies to investigate if allergenic structures in the inclusion complex are still accessible to IgE binding and whether the cyclodextrin can compete with IgE for free drug or sequester the bound drug from IgE-rocuronium complexes, a comparison of the association complexes of sugammadex and IgE antibody-rocuronium complexes has been proposed [118]. Sugammadex forms a stable complex with rocuronium with an association constant K_a_ of 1.8 × 10^7^ M^−1^. The average association constant of IgE-rocuronium complexes is not known and neither is/are the sensitizing antigen(s) of IgE antibodies that react with NMBDs. Although association constants for allergens such as multideterminant, multivalent pollen proteins are often high, e.g., K_a_ 10^10^ – 10^11^ M^−1^ [150, 151], the affinities and avidities of rocuronium-IgE complexes may be lower than first expected because of the bideterminancy of NMBDs and likely non-NMBD nature of the source(s) of the sensitizing agent(s) [103, 106]. This would lead to complexes of poorer ‘fit’ than the unknown sensitizing agent-IgE antibody complex. Higher affinities for the IgE-rocuronium complexes than for the sugammadex complex would result in the failure of sugammadex administration to mitigate a reaction; higher affinity of the sugammadex-rocuronium complex would result in sequestration of the offending drug and mitigation of anaphylaxis. Note also that affinities for antibodies reacting with the same hapten may differ by a factor of 10^3^ to 10^5^ [152]. If rocuronium-reactive IgE antibodies show such heterogeneity, sugammadex may mitigate an anaphylactic reaction in some patients but not others.

From the foregoing discussion, it is clear that sugammadex’s role in managing rocuronium-induced anaphylaxis is contentious. Reflecting this, ANZAAG of the Australian and New Zealand College of Anaesthetists (ANZCA) [153] advocates that, “The use of sugammadex in resuscitation of suspected anaphylaxis to rocuronium is not recommended”, although the Association of Anaesthetists of Great Britain and Ireland makes no such recommendation [154]. Given the current situation with data and arguments for and against the application of sugammadex for the rescue of anaphylaxis, the recommendation against its use may not be unreasonable and perhaps even prudent but it also seems too soon to discount many of the findings in the 23 case studies and to assume that host-guest sequestration and immune mechanisms involving IgE antibodies and mast cell receptors are not involved. Apart from the classical pathway involving IgE and its high-affinity receptor FcεRI, possible involvement of alternative pathways mediated by IgG, the low affinity FcγR locus, macrophages, platelet activating factor (PAF) and MRGPRX2 [137, 139, 155–157] should not be overlooked and the importance of nitric oxide, endothelial nitric oxide synthase, PAF, PI3K/Akt signalling, cytokines IL-4 and IL-13, sphingosine-1-phosphate and sphingosine kinases may be relevant [158, 159].

Recommended maximum non-irritant skin test concentrations of sugammadex are 100 mg/ml for prick testing and 10 mg/ml for intradermal testing [75].

### Antibiotics

In the early large Australian and French surveys (Section 4, Table 3), antibiotics accounted for 8.6% and 12.9%, respectively of drugs provoking immediate type I allergic reactions. β-Lactams were the dominant culprit antibiotics in both surveys with incidences of 86% and 88.5%, respectively but while reactions to cephalosporins dominated in Australian patients with an incidence approximately five times that of penicillins (73.1% to 15.4%), penicillins were implicated more often in the French survey (49% to 37% of reactions to antibiotics). By the time of the 2018 UK NAP6 survey [45], antibiotics were the dominant culprit drugs in the UK with involvement in 94 (67 definite and 27 probable reactions) of 192 cases. This predominance was also reported in some smaller surveys from the US and Europe [160–164]. Of the 94 cases in the NAP6 survey, amoxicillin-clavulanic acid accounted for 46 reactions (49%) and although teicoplanin made up only 12% of antibiotic administrations, it caused 36 reactions (38%). Further indications of teicoplanin’s increasingly high incidence of reactions [165] are seen in the figures per 100,000 exposures which were 8.7 for amoxicillin-clavulanic acid and 16.4 for teicoplanin. However, cephalosporins, particularly cefazolin [161–164, 166–168], are now the antibiotics most often implicated in allergic reactions in the perioperative period.

### Chlorhexidine

Since its introduction in 1954 as a disinfectant and antiseptic, chlorhexidine, a synthetic, stable, water-soluble cationic bisbiguanide (as the digluconate, dihydrochloride, or acetate salt), found wide and extensive usage in many everyday products, domestically and in medicine, industry, and the environment. With such broad human exposure, there was the potential for occasional adverse reactions in some individuals. Surprisingly, except for a few rare early reports [169, 170], it was not until the 1980s that hypersensitivities to chlorhexidene started to be well recognized, initially mainly in Japan and Australia [171–173] and then gradually more extensively. By the late 1990s after numerous reports of hypersensitivity reactions to chlorhexidine in Japan and warnings about its use on mucous membranes and wounds [173], the US Food and Drug Administration (FDA) issued an alert concerning chlorhexidine-induced anaphylaxis [174]. The first reported case of chlorhexidine anaphylaxis elicited via urethral exposure [175] was a forerunner of many similar reports which served to emphasize the importance of the route of exposure, particularly to unsealed wounds, mucous membranes, and impregnated central venous catheters, to the possibility of an allergic reaction including systemic anaphylaxis. In fact, besides transurethral, parenteral, wound, and mucous membrane exposure, reactions may be elicited topically, orally, rectally, vaginally, via the ophthalmic route, and even by inhalation [176].

Chlorhexidine’s widespread and often unrecognized presence in products, and the fact that it is not administered by anesthetists, at least partly explains why it has often been overlooked as a source of anaphylaxis in the perioperative setting. In that setting, it has recently been estimated to account for ~9% of hypersensitivities in each of the UK, Denmark, and Belgium [45, 167, 177]. In the NAP6 audit project, the incidence of reactions was 0.78 per 100,000 exposures, the third most common cause of ADRs [45]. These figures are strikingly different to results from the early surveys where chlorhexidene is not mentioned. Reactions to chlorhexidine may be immediate, which are most common, or delayed, both reactions are known to occasionally occur in the same patient, and severity can range from mild skin reactions to life-threatening angioedema and anaphylaxis [178–180]. Reactions may occur during surgery but also in the postoperative period and, in up to 80% of patients with a reaction, anaphylaxis may be life-threatening [180]. A survey of chlorhexidene induced anaphylaxis in surgical patients found exposure to urinary catheter lubricant, chlorhexidene-coated central venous catheters, and topical antiseptic solutions were the most common sources of allergic sensitization [179]. Immediate allergic reactions after parenteral exposure usually appear within a few minutes and up to an hour after wound or mucosal exposure. Contact-induced sensitization may manifest as allergic contact dermatitis and stomatitis. Patch testing 7,610 patients in Finland with 0.5% chlorhexidene revealed positive reactions in 0.47% of patients [181]. Allergic reactions to chlorhexidine have been reported in the workplace, especially amongst healthcare workers but it is likely that the true prevalence of occupational cases is underestimated [182]. Five percent of 92 healthcare workers in Thailand responded with cutaneous rashes when exposed to chlorhexidene digluconate 2% and 5% [183].

Type I immediate reaction to chlorhexidene was early demonstrated to be mediated by IgE-antibody [172, 184]. An immunoassay developed in the author’s laboratory showed good specificity and high sensitivity and proved to be a useful diagnostic tool. Structure-activity studies of the antigen-antibody interaction with respect to the features of the complementary structures recognized by the antibody combining site remains one of the best-defined drug allergies at the molecular level [185]. Subsequently, a commercial immunoassay with high sensitivity (84.2%) and specificity (93.7%) was developed and shown to be a reliable diagnostic method subject to cautionary evaluation in the presence of high total IgE levels [186]. As with other drug allergies, diagnosis is aided by employment of other in vitro tests (including the BAT [187] and histamine release assays) and in vivo tests (skin prick and intradermal tests and patch tests for delayed reactions). To help anticipate perioperative allergic reactions, patient histories relevant to chlorhexidene along with specific tests are now undertaken routinely in some countries [45, 75, 177, 186]. Recommended skin test concentrations for chlorhexidene are 5 mg/ml (0.5%) for skin prick tests and 0.002 mg/ml (0.0002%) for intradermal testing [74, 75, 188]. With lack of exposure, skin test positivity declines with time.

### Water soluble blue dyes

Water-soluble blue dyes, with and without isotope (e.g., technetium-99 m colloid), selectively localized into lymphatics, are increasingly used for diagnostic purposes particularly the identification of sentinel lymph nodes in melanoma patients and in other cancers including breast, cervical, bladder, and endometrial cancer. The triarylmethane dyes, patent blue V and isosulfan blue (Lymphazurin^®^), are most often used, the former in the UK and the latter in the US. Methylene blue, a thiazine dye, is substantially cheaper but is not always approved for sentinel lymph node localization [8, 189]. Allergic/adverse reactions to patent blue V and isosulfan blue, known for over 60 years, have a reported incidence between 0.07% and 2.7% with a mean of 0.71% [190]. Symptoms range from mild, e.g., erythema and urticaria, to severe and life-threatening hypotension, pulmonary edema, and vascular collapse. Reactions to blue dyes in the perioperative setting, including anaphylaxis, and in particular patent blue V, have been increasingly recognized in recent years [8, 189], for example, in the NAP6 survey in the UK, patent blue V, with an incidence of 1 in 7,000, was the fourth most recognized culprit drug. By comparison, incidences of the other three main offenders were antibiotics 1 in 27,000, NMBDs 1 in 19,000, and chlorhexidene 1 in 127,500 [45].

#### Patent blue V

Allergic reactions may occur following injection of patent blue V in the procedure of sentinel lymph node biopsy (SLNB) for the detection of cancer cells in women with operable breast cancer. Data on side effects of patent blue V were collected in a UK-wide SLNB NEW START training program and the Axillary Lymphatic Mapping Against Nodal Axillary Clearance (ALMANAC) multiccnter trial under the auspices of the Medical Research Council of the UK [191, 192]. Adverse reactions were seen in 72 of 7,917 (0.91%) patients with breast cancer [193]; 4 patients (0.05%) had non-allergic reactions; 23 patients (0.29%) experienced grade I allergic skin reactions (urticaria, blue hives, pruritus, or generalized rash); 16 (0.2%) had grade II reactions (transient hypotension/bronchospasm/laryngospasm); and 5 patients (0.06%) developed severe grade III reactions (severe hypotension requiring vasopressor support, change or abandonment of planned procedure, and/or high dependency unit (HDU)/intensive therapy unit (ITU) admission).

A retrospective study of a database of 1247 patients who reacted to patent blue V over a 2-year period 2008–2010, revealed 11 patients (0.88%) who experienced immediate hypersensitivity reactions during anesthesia. Six patients (0.48%) had minor grade I reactions (urticaria, blue hives, pruritis or generalized rash), 4 (0.32%) had grade II reactions (transient hypotension/bronchospasm/laryngospasm), and 1 patient (0.08%) experienced a grade III reaction (hypotension requiring prolonged vasopressor support). Time of reaction onset, which often coincided with the induction of anesthesia, ranged from 10–45 min, 7 cases (63.6%) were cancelled or postponed, and no fatalities occurred [194]. Three patients who had systemic reactions including hypotension and rash following injection of patent blue V for SLNB, each tested skin test-positive to patent blue V but also to methylene blue, demonstrating cross-sensitivity between the dyes [195].

Symptoms usually occur within a few min to 45 min after injection manifesting as shock (including bronchospasm and gastrointestinal symptoms), characteristic large blue-green hives, or so-called ‘blue urticaria’, blue-colored periorbital angioedema, angioedema of hands and arms, erythema, and pruritus [196]. Although some features of reactions suggest direct mast cell activation, positive skin tests, passive transfer of the sensitivity, and detection of specific IgE antibodies indicate that reactions are generally type I hypersensitivities [196–198]. Elevated tryptase levels in patients’ sera support diagnoses of anaphylaxis [196, 199]. Both IgE assays [197] and BAT [200] are a useful aid for diagnosis but their limited availability restricts their routine use and, in any case, skin testing has proved a reliable diagnostic procedure.

#### Isosulfan blue

Several US surveys have assessed the incidence of isosulfan blue in allergic/adverse reactions. A combination of studies totaling 8,372 patients revealed 119 reactions (1.42%). For severe reactions (grade III), the percentages were 0.44% for isosulfan blue and 0.06% for patent blue V, respectively [193]. Results from five individual surveys revealed allergic/adverse reactions in the range 0.7–1.9% [201] while adverse events associated with the intraoperative injection of isosulfan blue occurred in 28 of 1,835 patients (1.5%) [202]. Potentially life-threatening hypotension occurred in 14 patients, skin reactions in 21, and edema in 1 patient. Importantly, onset of reactions occurred over a wide time range (1–180 min), in some cases with a long reaction duration. Diagnostic methods include skin tests (see below), assay for IgE antibodies, and tryptase determinations [203–205].

#### Methylene blue

Methylene blue has been used for sentinel lymph node localization but it is not always approved for that purpose. With the aim of assessing the suitability and accuracy of SLNB mapped with methylene blue alone in breast cancer patients, Li et al. [206] undertook a review and meta-analysis of 18 studies to determine the identification rate and false negative rate of sentinel node biopsy in breast cancer. Although the analysis showed that mapping with methylene blue alone provides an acceptable identification rate, the false negative rate is excessive, indicating that caution is warranted when using the dye alone. Reported adverse effects resulting from its use include skin necrosis and subcutaneous ulcers, rarely seen anaphylaxis, pulmonary edema, spinal cord necrosis, and phototoxicity [189, 207–211]. Immediate hypersensitivity reactions to methylene blue are rare although several cases of allergic reactions following treatment with methylene blue-treated fresh frozen plasma were reported from France [212].

#### Summary of diagnosis of blue dye hypersensitivity

For patent blue V, isosulfan blue, and methylene blue, a 1:100 dilution of the stock solution (1%) is generally suitable for intradermal testing [74]. Skin test guidelines issued by ANZAAG are: Patent blue V — prick testing, 25 mg/ml undiluted; intradermal testing, initial 1–1,000 dilution (25 μg/ml), final 1–100 dilution (250 μg/ml), maximum 2.5 mg/ml. Isosulfan blue 10 mg/ml, — prick testing 1–10 (1 mg/ml) and undiluted; intradermat testing, initial 1–1000 dilution (10 μg/ml), then 1–100 (100 μg/ml), and final dilution 1–10 (1 mg/ml). Methylene blue — prick testing, 10 mg/ml, undiluted; intradermal testing, initial 1–1,000 (10 μg/ml), final 1–100 dilution (100 μg/ml), maximum 100 μg/ml [75].

Immunological cross-reactivity between patent blue V and isosulfan blue, is well known [213] especially in skin testing, but methylene blue is generally thought to be non-cross-reactive (but compare Keller e al. [195]). As discussed above, in recent years there has been a significant increase in perioperative cases of anaphylaxis to blue dyes, reflected for example in results of the first multicenter survey of anaphylaxis during general anaesthesia in the UK, 2005–12 [44] and the 2018 UK NAP6 [45] surveys. Positive skin tests and some IgE antibody studies suggest an IgE antibody-mediated mechanism of reactions but there may be more than one mechanism, for example, direct actions on mast cells and basophils. An importance for diagnosis and treatment is the observation of a large time gap (~ 30 min) between dye injection and symptom onset and the need in some patients for prolonged (several hours) epinephrine treatment [199]. Biphasic anaphylactic reactions have been reported for both patent blue V and isosulfan blue [213–215].

### Colloids

#### Gelatin

Gelatin as a blood volume expander is marketed as Haemaccel^®^, a cross-linked preparation with urea, MW ~ 35,000 Da and Gelofusine^®^, which is succinate-linked with a mean MW ~ 30,000 Da. Use of IV gelatin has increased in recent years due to safety concerns with starch-based colloids. Gelatin carries the highest incidence of anaphylaxis and is more likely to cause anaphylaxis than albumin and other colloids [216–218], Allergic reactions to gelatin colloids occur with symptoms of anaphylaxis, sneezing, bronchospasm, and urticaria. Relative to other drugs used in anesthesia, the incidence of reactions to gelatin is equal to that of rocuronium at 6.2 per 100,000 administrations [45]. A survey of 19,593 patients in France of anaphylactoid reactions to colloid plasma substitutes 48.1% of whom were given gelatin, revealed a reaction incidence of 0.345% [216]. In a recent retrospective review of 12 patients with severe anaphylaxis to gelatin-based solutions [219], 3 reacted within 5 min of administration while 6 reacted 10–70 min later, an unusual similar time course seen with anaphylaxis to chlorhexidine and blue dyes [45]. The most common symptoms were hypotension, cutaneous signs, tachycardia, and bronchospasm. Three patients suffered cardiac arrest. Allergic reactions in 11 patients were confirmed by skin testing and by IV provocation in one patient. Serum tryptase levels were elevated in all patients. Skin prick test concentrations were succinylated gelatin undiluted and 1:10 dilution; intradermal solutions were 1:10,000 starting dose to undiluted. Undiluted solutions were shown to be non-irritant in 10 control subjects. Usually recommended skin test concentrations for Gelofusine^®^ and Haemaccel^®^ are 35 mg/ml for prick testing and dilutions of from 1 in 1,000 to 1 in 10 for intradermal testing. The authors concluded that given the risk of severe allergy and absence of evidence of clinical benefit, the use of gelatin-based solutions in the perioperative setting should be reassessed [219].

#### Hydroxyethyl starch

In the French study mentioned above [216], the incidence of reactions to starch-based colloids was 0.058%. The relative risk of anaphylactoid reactions to starches compared to gelatins was sixfold less and less than dextrans and albumin for adverse reactions. Adverse reactions to hydroxyethyl starch (HES) include anaphylaxis, erythema, urticaria and, in particular, pruritus, the latter occurring with an incidence of up to 40%, Pruritus is usually so severe it has a major impact on patients’ quality of life [220]. All HES solutions, regardless of different molecular weights and substitution, are generally refractory to treatments, resulting in pruritus persisting for up to 2 years. The underlying mechanism of pruritus appears to be tissue deposition of HES mainly in macrophages. On the basis that the risks of HES outweigh the benefits, in 2013, the Pharmacovigilance Risk Assessment Committee of the European Medicines Agency (EMA) recommended that the marketing authorization of HES infusions be withdrawn. On the recommendation of the UK Commission on Human Medicines, HES preparations were also withdrawn from the UK. Although HES has not been withdrawn completely in the US, the US FDA recommended that it should not be used in critically ill patients or in those with pre-existing renal dysfunction [221].

#### Dextrans

Dextrans are polysaccharides composed of *D-*glucose units linked α-(1-6) with branches linked α-(1-3). Two intravenous solutions containing the high molecular weight dextrans 40 and 70 are used for plasma volume expansion. Dextran-induced anaphylactic reactions (DIAR) range in severity from mild erythema (grade I) to death (grade V). Reactions are caused by pre-existing circulating antibodies to dextran, mainly IgG, forming immune complexes with the injected dextran. Dextran 1 (molecular weight 1,000 Da), administered as a hapten immediately before dextran 40, inhibits the formation of immune complexes and subsequent hypotension and produces a 35-fold reduction in the incidence of severe DIAR. With an incidence of anaphylactoid reactions of 0.273% [216], dextrans 40 and 70 are now the safest of all the volume expanders in clinical use. Since dextran antibodies cross the placenta and cases of neurological impairment and death can occur in neonates, dextran should not be administered to pregnant women. Skin test concentrations for diagnosis are dextran 6–10 mg/ml for prick testing and up to a 1 in 100 dilution of this solution for intradermal testing.

### Drugs more rarely involved in preoperative, perioperative, and postoperative adverse reactions

**Opioids** may provoke respiratory depression [222] and ST [14] (Section 2) but are rarely involved in type I IgE antibody/FcεRI-mediated allergic responses although cutaneous wheal and flare reactions and some hemodynamic effects of histamine-releasing opioids such as morphine, codeine, and meperidine may lead to an anaphylactoid response and false diagnoses of an IgE-mediated reaction [8, 223–225]. The histamine releasing effect can affect the reliability of skin testing although this can be successfully undertaken by using suitably diluted test solutions, for example, 1 mg/ml of morphine for prick testing and 5–10 μg/ml intradermally [8, 226]. Skin tests with suitable concentrations of morphine complemented with the tryptase determination, a reliable immunoassay for morphine plus suitable inhibition studies, and/or BAT, can lead to a confident diagnosis [8, 108, 226–229]. Involvement of MRGPRX2 in a morphine-induced reaction might be expected but for this receptor and an IgE/FcεRI-mediated reaction the clinical presentation is the same [137]. It should be noted that opioids do not feature prominently in many of the surveys of perioperative drug reactions, in fact, there is no mention of them in the NAP6 report [45].

**Heparin and related agents** used medicinally range from unfractionated polymers with molecular weights in the range 12–20 kDa to low molecular weight (LMW) heparins (4–6 kDa) that include, amongst others, **dalteparin** [230] and **enoxaparin** [231]. Often administered to patients during cardiac surgery including pulmonary bypass surgery and for acute coronary syndrome, atrial fibrillation, deep vein thrombosis, and pulmonary embolism, heparin acts an anticoagulant, preventing clots by binding to antithrombin III and inactivating thrombin and factor X_a_. The incidence of adverse reactions to heparins is ~0.2%. The most common reactions are thrombocytopenia [232], anaphylactoid reactions and immediate hypersensitivity including anaphylaxis [233], skin necrosis, some cutaneous reactions, and a few delayed reactions. In thrombocytopenia, heparin binds platelet factor 4 (PF4) on the platelet surface and this complex in turn binds IgG, an antibody common after heparin administration. The resultant complex causes the release of microparticles that promotes thrombin formation. Classified as a type II cytotoxic hypersensitivity response, the formation of immune complexes on the platelet surface also suggests a type III mechanism. Up to about 50% of heparin-treated patients may form antibodies reactive with the heparin-PF4 complex.

Besides unfractionated heparin, a large array of other anticoagulants find common usage [234]. In addition to the LMW heparins, these include the semisynthetic **heparinoids** like **danaparoid**; the naturally occurring polypeptide **hirudin** and recombinant forms **desirudin** and **lepirudin** [235]; **fondaparinux**, a synthetic pentasaacharide with structural identity to a sequence of five sugar units of heparin; **pentosan polysulfate**, is a semi-synthetic heparin-like polysulfated xylan; and synthetic direct inhibitors of thrombin, **argatroban** and **dabigatran**.

Skin test concentrations for both heparins (heparin sodium, **nadroparin**, dalteparin, enoxaparin) and heparinoids (danaparoid, fondaparinux) in the ENDA/EAACI guidelines are undiluted for prick testing, 1–10 dilutions for intradermal testing, and undiluted for patch testing [236].

Some **polypeptides** have a history of anaphylaxis related to their use in anesthesia and surgery. The incidence of allergic sensitivity to natural rubber **latex** in the population is estimated to be 2.1–3.7% but it can be higher in certain groups, e.g., dentists and spina bifida patients. Beginning in the 1980s and extending into the 1990s, the number of reports of anaphylaxis due to latex increased alarmingly, in some surveys reaching up to ~20% of all perioperative cases of anaphylaxis (Table 3) Increased awareness and changes plus widespread adoption of measures to institute latex-free protocols in operating, treatment and recovery rooms alleviated the situation such that cases of anaphylaxis to latex in recent surveys are now rare [45].

**Protamine** is used to reverse the anti-coagulant effect of heparin during cardiac catheterization and cardiopulmonary bypass. It provokes a number of adverse effects including flushing, rash, urticaria, angioedema, wheezing, hypotension, bronchospasm, cardiovascular collapse, and sometimes death. Protamine releases histamine and tryptase from human basophils, heart mast cells and synovial mast cells but not from lung mast cells [237]. Incidence rates of protamine reactions in patients undergoing cardiopulmonary bypass range from 0.1% to 13% [238, 239] while mortality is estimated at 2% [240]. Insulin-dependent diabetics show a higher incidence of anaphylaxis to protamine than patients not receiving insulin [241], suggesting sensitization by protamine in neutral protamine Hagedorn (NPH)-insulin preparations. Protamine intradermal skin tests show poor specificity with false positives and irritant responses in normal controls. Both skin and antibody tests have proved unsuitable for screening patients before administration of protamine.

**Aprotinin**, a serine protease inhibitor from bovine lung, promotes fibrinolysis, reduces thrombin generation, and maintains platelet function, accounting for its use in cardiac surgery, organ transplantations and hip surgery where reductions in bleeding, blood loss and transfusion needs are important. Anaphylactic reactions to aprotinin are almost invariably seen after previous exposure to the drug [242]. Aprotinin’s protein nature and bovine origin can lead to the production of IgG and IgE antibodies as well as cases of anaphylaxis. Analysis of aprotinin-induced anaphylaxis in over 12,000 patients exposed to the drug in cardiac surgery, revealed hypersensitivity reaction incidences of 4.1%, 1.9% and 0.4% in less than 6 months, 6–12 months, and more than 12 months re-exposure intervals, respectively [243]. Skin test concentrations used are 10,000 IU/ml in prick tests and up to a maximum of 100 IU/ml intradermally.

**Ondansetron**, a selective 5-HT_3_ serotonin antagonist used as an antiemetic is known to provoke both IgE-antibody- and non-IgE-mediated reactions including cases of anaphylaxis which are uncommon [244–246]. For skin testing, the drug is used at a concentration of 2 mg/ml for the prick test and 0.02 mg/ml for intradermal testing. Control subjects proved positive at concentrations of 0.2–2 mg/ml (compare concentrations in [244, 245]).

## Outlook and some important developments

ADRs are influenced by, obviously first and foremost, drugs, but also by a wide range of factors including age, sex, ethnicity, patient pathologies, route of administration, drug interactions, and importantly, genotype. The importance of the latter factor is being increasingly realized although application of pharmacogenomics to ADRs seen during anesthesia and surgery continues to be hampered for a number of reasons including lack of education and training of health care professionals; the still limited knowledge of the pathophysiology of many ADRs and drug pharmacogenomics; the need for wider genotyping and understanding of its benefits; the importance of pharmacogenomic drug labeling; and need for more guidance from regulatory authorities. Although an extended consideration of the place of pharmacogenomics in drug reactions experienced in the peri- and postoperative periods is beyond the scope of the present review, attention is drawn to the increasingly recognized associations of CYP enzymes and HLA genotypes with an increasing number of disease states [247]. By way of examples, are the associations of HLA genotypes with cutaneous ADRs (Table 1) and CYP enzyme associations with analgesics (Table 2), tramadol with CYP2D6, and the anticoagulant prodrug clopidogrel with CYP2C19. Clopidogrel is a good example to consider here. CYP2C19 converts the prodrug to its active metabolite but some individuals are poor metabolizers (PM) while others may be intermediate metabolizers (IM). PM individuals have two non-functioning copies of the *CYP2C19* gene while IM individuals have one non-functioning and one functional *CYP2C19* gene. Individuals of CYP2C19 PM phenotype do not therefore receive the full antiplatelet effect while the effect is diminished in CYP2C19 IM individuals. Ethnicity plays a part, for example, only 2% of Caucasians and 4% of African American are PMs but the figures for Chinese and Oceanians are 14% and 57%, respectively. In 2022 the FDA issued a boxed warning on the diminished antiplatelet action of clopidogrel in CYP2C19 patients and the Clinical Pharmacogenetics Implementation Consortium (CPIC) recommended substitute drugs such as ticagrelor or prasugrel for patients with a number of different conditions including acute coronary syndrome [248]. A similar situation exists for patients taking tramadol where CYP2D6 converts the prodrug to its active metabolite and different allelic variants produce different enzyme functions from no, to normal, to increased activity [25–27] (Section 3). The anticoagulant warfarin provides another interesting pharmacogenomic example of an ADR. Greek Hellenes and Greek Cypriots show differences in allele frequency of *VKORC1* compared to other Caucasians, Africans, and Asians, and no differences in CYP2C9 and CYP3A5 allele frequencies compared to Caucasians but significant differences when compared with Asians and Africans. About 50% of Greek Cypriots carry at least two risk alleles associated with warfarin sensitivity and a potential high risk of bleeding after normal doses of anticoagulants [249].

The above considerations of ADRs and pharmacogenomics emphasize the need for clinicians to know the relevant genotype when prescribing some drugs, to understand the pathophysiology of the life-saving and life-prolonging drugs they prescribe, and for the safe management of patients.

Although NMBDs and antibiotics have remained as the main culprit drugs provoking anaphylaxis during anesthesia since the earliest sufficiently large, and well conducted and executed surveys (Table 3), recent findings [45] of the increased incidence of anaphylaxis to blue dyes and antibiotics, in particular teicoplanin, demonstrates the need for vigilance in the increasing use of some previously generally unrecognized sources of severe reactions. By contrast, the spectacular increase in the number of cases of latex-induced anaphylaxis in the decade 1980–1990, rapidly declined following recognition of the increasing use of rubber-based materials, particularly rubber gloves, and the introduction of latex-free protocols in operating, treatment, and recovery rooms [8].

Following the early years of colloid use, there has been a reassessment by some clinical investigators and regulatory agencies of the risks associated with their use, in particular the protein gelatin (usually cow or pig) due to increasing reports of allergic reactions, including anaphylaxis [217–219], and hydroxyethyl starch for severe, protracted pruritus refractory to treatment and the risk of kidney injury [220, 221]. Again, these developments emphasize the need to remain aware of changed recommendations based on extended experiences with drug usage.

Recent research on the immune response to heparin adds a new and interesting aspect to our knowledge of antibodies to the anticoagulant. As discussed above (Section 5.8), in thrombocytopenia, heparin binds platelet factor 4 (PF4) on the platelet surface and together with anti-heparin IgG, the resultant complex promotes thrombin formation. With the recent interest in vaccine-induced immune thrombotic thrombocytopenia (VIITT) caused by anti-PF4 antibodies activating platelets and the finding of these antibodies in patients infected with Covid-19, concerns were raised that antibodies induced by vaccination might cause thrombosis by cross-reacting with PF4. SARS-CoV-2 spike protein and PF4 share a similar epitope(s). Investigations revealed that immune responses to PF4 and the SARS-CoV-2 spike protein are independent and antibodies in patients with VIITT and thrombosis do not cross-react with the spike protein indicating that the immune response to the spike protein does not induce VIITT [250].

Despite a long-standing belief by many, opioids are rarely involved in type I IgE antibody/FcεRI-mediated allergic responses and this is reflected in the more recently published surveys of perioperative drug-induced anaphylaxis (Sections 4 and 5.8). There are few reports of anaphylaxis to opioids [8, 251]; most of the reactions elicited by morphine for example, are pruritus, urticaria, and pain with the involvement of released histamine and activation of the mast cell receptor MRGPRX2 [137, 252, 253] (Section 5.3).

Clinicians should be aware of the similarity between drug-induced true allergic type I IgE/FcεRI- and pseudoallergic MRGPRX2-mediated ADRs, the clinical features of each, and their distinguishing characteristics [8, 137, 252–254]. Activation of MRGPRX2 provokes responses clinically similar to IgE/FcεRI-mediated reactions of itch, inflammation, and pain without the involvement of antibody priming. A negative skin test does not necessarily preclude MRGPRX2 involvement and the absence of specific IgE antibody tests for many of the drugs that activate both receptors also makes it difficult to distinguish the two reactions [137, 252, 253, 255]. In addition, some agents, for example, tetrahydroisoquinolone NMBDs and fluoroisoquinolone antibiotics may activate both receptors [137, 253]. Ruling out the involvement of any other nonimmune mechanism and immune processes but particularly IgE/FcεRI-mediated degranulation of mast cells, has been suggested as a way of overcoming the problems of confidently identifying MRGPRX2 activation and diagnosing resultant pseudoallergic reactions [255]. However, such a process of exclusion is, at best, a fall-back approach to the preferred methodology of direct implication of MRGPRX2 activation.

As for most GPCRs, MRGPRX2 signals via β-arrestin [256], and this property can be employed in the form of a β-arrestin recruitment assay [138, 257–259] to aid diagnosis by discriminating between pseudoallergic MRGPRX2- and true allergic IgE-mediated reactions. Following a clinical assessment which includes a tryptase determination, skin and IgE testing are undertaken. The BAT, mast cell activation test (MAT), and T-lymphocyte activation test (TAT) [255] may be used to check negative skin and IgE tests or in the absence of IgE assays. Using *MRGPRX2-*transfected cells (e.g., HEK293 or CHO-K1) [138, 254, 257–259], MRGPRX2 activation is assayed for both β-arrestin (G-protein-independent) and Ca^2+^ (G- protein-dependent) endpoints [259] and results compared to a reference histamine release assay or other degranulation assay (e.g., β-hexosaminidase assay or flow cytometric measurement of cell surface CD107 and CD63) [138, 255, 259]. Note that some ligands activate one (e.g., G-protein biased icatibant), or both (G- protein-dependent and independent), pathways (e.g., compound 48/80) [138, 260].

The stepwise application of skin and specific IgE antibody tests together with MRGPRX2 activation and histamine assays provides a sensitive and rapid approach for distinguishing pseudoallergic from true allergic reactions and identifying agonists. The methodology also provides the means to investigate the safety of known and newly introduced small molecule drugs as well as biologically active peptides [138, 259].

## Data Availability

Data is available from author on request.

## Abbreviations

ADR: Adverse drug reaction
AGEP: Acute generalized erythematous pustulosis
ANZAAG: Australian and New Zealand Anaesthetic Allergy Group
ANZCA: Australian and New Zealand College of Anaesthetists
BAT: Basophil activation test
CNS: Central nervous system
CYP: Cytochrome P450
DIAR: Dextran-induced anaphylactic reaction
DILI: Drug-induced liver injury
DRESS: Drug reaction with eosinophilia and systemic symptoms
EAACI: European Academy of Allergy and Clinical Immunology
ENDA: European Network of Drug Allergy
EMA: European Medicines Agency
FDA: US Food and Drug Administration
GPCR: G-protein-coupled receptor
HES: Hydroxyethyl starch
HDU: High dependency unit
HLA: Human leukocyte antigen
ICU: Intensive care unit
ITU: Intensive therapy unit
MAT: Mast cell activation test
MRGPRX2: Mastocyte-related G-protein-coupled receptor X2
NAP: National Audit Projects
NHS: National health service
NMBD: Neuromuscular blocking drug
NSAID: Non-steroidal anti-inflammatory drug
PAF: Platelet activating factor
PF4: Platelet factor 4
SLNB: Sentinel lymph node biopsy
TAT: T-lymphocyte activation test
TEN: Toxic epidermal necrolysis
VIITT: Vaccine-induced immune thrombotic thrombocytopenia
VKORC1: Vitamin K epoxide reductase complex subunit 1
